# Hepatointestinal complications in polycystic kidney disease

**DOI:** 10.18632/oncotarget.20901

**Published:** 2017-09-15

**Authors:** Shih-Ting Huang, Ya-Wen Chuang, Tung-Min Yu, Cheng-Li Lin, Long-Bin Jeng

**Affiliations:** ^1^ Division of Nephrology, Taichung Veterans General Hospital, Taichung, Taiwan; ^2^ Graduate Institute of Public Health, China Medical University, Taichung, Taiwan; ^3^ Graduate Institute of Clinical Medical Science and School of Medicine, College of Medicine, China Medical University, Taichung, Taiwan; ^4^ Management Office for Health Data, China Medical University Hospital, Taichung, Taiwan; ^5^ College of Medicine, China Medical University, Taichung, Taiwan; ^6^ Department of Surgery, Organ Transplantation Center, China Medical University Hospital, Taichung, Taiwan

**Keywords:** bleeding, cholangitis, cirrhosis, pancreatitis, polycystic kidney disease

## Abstract

**Background:**

The objective of this study was to determine the incidence of major hepatointestinal complications in patients with polycystic kidney disease (PKD).

**Methods:**

We analyzed the Taiwan National Health Insurance claims data (2000–2010) of 6031 patients with PKD and 23,976 non-PKD hospitalized controls. The control cohort was propensity score matched with the PKD cohort at a 1:4 ratio. All patients were followed up from the index date to the first inpatient diagnosis of hepatointestinal complications, death, or 31 December, 2011. Cox proportional hazard regression models were used to identify the risk of outcome after adjustment for potential confounders.

**Results:**

The incidence rates of acute pancreatitis, cholangitis, peptic ulcer bleeding, and cirrhosis were 5.72, 4.01, 19.9, and 5.46 per 1000 person-years, respectively, in the PKD cohort. Compared with the non-PKD controls, patients with PKD exhibited an increased risk of hospitalization for acute pancreatitis, cholangitis, peptic ulcer bleeding, and cirrhosis (adjusted subhazard ratio [aSHR]: 2.36, 95% confidence interval [95% CI], 1.95–2.84]; 2.36, [95% CI, 1.95–2.84]; 2.41, [95% CI, 1.93–3.01]; 2.41, [95% CI, 2.17–2.67]; and 1.39, [95% CI, 1.16–1.66], respectively; all *p* < 0.001). PKD, chronic kidney disease, and alcoholism were independent predictors of all these hepatointestinal complications. Kaplan–Meier analysis revealed an increased overall mortality in patients with PKD who developed acute pancreatitis and peptic ulcer bleeding (log-rank *p* < 0.05).

**Conclusion:**

PKD is associated with clinically significant extrarenal complications including acute pancreatitis, cholangitis, peptic ulcer bleeding, and cirrhosis.

## INTRODUCTION

Polycystic kidney disease (PKD) is the most common hereditary renal disease and the fourth most common cause of end-stage renal disease (ESRD) [1, 2]. Although most patients exhibit progressive renal injury, the average age of onset of ESRD and survival rates in ESRD have increased [3]. The major causes of death are cardiovascular diseases, infection, and central nervous system disorders; whereas uremia was the cause of death in only 2.2% of the patients [4]. Thus, the contribution of extrarenal manifestations such as liver cysts and cardiac disease to morbidity and mortality are increasingly recognized [5].

Among patients with autosomal dominant PKD (ADPKD), approximately 75%–90% have associated polycystic liver cysts (PLD) [6]. Polycystic liver disease (PLD) persists after ESRD and is a major feature in patients with PKD. PLD accounts for 10% of deaths in patients with ADPKD on hemodialysis [7]. Considerable improvements in radiological and surgical managements for cystic complications have been made in PKD patients with PLD [8]. However, more than 60% of patients with ADPKD present with abdominal pain, which has a detrimental effect on quality of life (QOL) [9, 10]. Anemia, low serum albumin level, ascites, and abdominal distention related to total liver and kidney volumes have a significantly negative influence on the QOL of patients with PKD [11]. Thus, the management of abdominal symptoms is important for improving the QOL of patients with PKD.

Abdominal pain and distention remain a diagnostic challenge in patients with PKD because renal and nonrenal sources of pain unrelated cystic complications must be considered [8]. Acute and chronic intra-abdominal complications such as inflammation, infection, and bleeding may manifest abdominal symptoms in PKD; this phenomenon has not been fully evaluated. The present study investigated the risk and outcome of hepatointestinal complications, including acute pancreatitis, cholangitis, peptic ulcer bleeding, and cirrhosis, beyond the conventional extrarenal manifestations in patients with PKD.

## RESULTS

### Baseline characteristics

Baseline demographic and comorbid characteristics of the PKD cohort were compared with those of the propensity-score (PS)-matched cohort (Table 1). The mean age of the PKD cohort was 58.2 (SD: 17.0) years, and 3398 (56.3%) were men. Chronic kidney disease (CKD) was prevalent (32.4%), and 30.9% of the patients in the PKD cohort received maintenance dialysis. No significant differences in baseline characteristics were observed among the two cohorts.

**Table 1:** Demographic characteristics and comorbidities of patients with and without polycystic kidney disease (propensity score-matched cohort)

	Polycystic kidney disease				
	Yes(N=6031)		No(N=23976)		
	n	%	n	%	p-value
Age, year					0.99
20-49	2063	34.2	8199	34.2	
50-64	1689	28.0	6718	28.0	
≥ 65	2279	37.8	9059	37.8	
Mean (SD)^#^	58.2	17.0	57.9	17.0	0.19
Gender					0.98
Female	2633	43.7	10471	43.7	
Male	3398	56.3	13505	56.3	
Comorbidity					
CKD	1953	32.4	7688	32.1	0.64
ESRD	1862	30.9	7336	30.6	0.68
GB stone disease	438	7.26	1681	7.01	0.50
Hyperlipidemia	591	9.80	2295	9.57	0.59
Obesity	11	0.18	27	0.11	0.17
Diabetes	1110	18.4	4382	18.3	0.82
Alcoholism	66	1.09	233	0.97	0.39
Chronic HBV infection	213	3.53	790	3.29	0.36
Chronic HCV infection	202	3.35	754	3.14	0.99
NAFLD	170	2.82	643	2.68	0.56

Chi-square test; ^#^: T-test.

HBV: Hepatitis B virus; HCV: Hepatitis C virus; NAFLD: Nonalcoholic fatty liver disease.

### Primary outcomes

The mean follow-ups for patients who developed acute pancreatitis, cholangitis, peptic ulcer bleeding, and cirrhosis in the PKD cohort were 4.64 ± SD: 3.58, 4.67 ± SD: 3.57, 4.43 ± SD: 3.57, and 4.57 ± SD: 3.57 years, respectively. Figure 2 presents the risks of acute pancreatitis, cholangitis, peptic ulcer bleeding, and cirrhosis in terms of the Kaplan–Meier survival analysis for the PS-matched cohort followed up for more than 10 years. The risks were significantly higher in the PKD cohort than in the control cohort (log-rank test, *p* < 0.001). Notably, the incidence of peptic ulcer bleeding was more prevalent than that of acute pancreatitis, cholangitis, and cirrhosis through the follow-up period.

During follow-up, 160 patients developed acute pancreatitis in the PKD cohort (Table 2). The incidence rate of acute pancreatitis was higher in the PKD cohort than that in the control cohort (5.72 vs 2.37 per 1000 person-years), with an incidence rate ratio (IRR) of 2.41. In the multivariate model, the adjusted subhazard ratio (aSHR) of acute pancreatitis was 2.36-fold higher in the PKD cohort (95% confidence interval [CI], 1.95–2.84, *p* < 0.001) than that in the control cohort.

**Table 2:** Incidence and subhazard ratio of hepatointestinal complications for patients with and without polycystic kidney disease calculated in a competing risk (death) model

	Polycystic kidney disease							
	Yes			No				
Outcome	Event	PY	Rate^#^	Event	PY	Rate^#^	Crude SHR^†^ (95% CI)	Adjusted SHR^‡^ (95% CI)
Acute pancreatitis	160	27972	5.72	302	127285	2.37	2.37 (1.97, 2.86)***	2.36 (1.95, 2.84)***
Cholangitis	113	28176	4.01	188	127621	1.47	2.27 (1.82, 2.83)***	2.41 (1.93, 3.01)***
Peptic ulcer bleeding	532	26700	19.9	1068	125502	8.51	2.42 (2.18, 2.68)***	2.41 (2.17, 2.67)***
Cirrhosis	154	28183	5.46	501	127143	3.94	1.41 (1.18, 1.69)***	1.39 (1.16, 1.66)***

PY, person-years; Rate#, incidence rate per 1000 person-years.

Crude SHR†, relative subhazard ratio.

Adjusted SHR‡: Covariables found significantly associated with outcome diseases in the univariable competing risk (death) model were further examined using the multivariable competing risk (death) model.

***p < 0.001.

In the PKD cohort, 113 patients developed cholangitis with an incidence rate of 4.01 per 1000 person-years. The IRR was 2.72, and the PKD cohort had a 2.41-fold higher risk of cholangitis (95% CI, 1.93–3.01) than the control cohort. In an analysis of risk factors, alcoholism, gallstones, PKD, CKD, and increasing age were independently associated with acute pancreatitis and cholangitis (Table 3).

**Table 3:** Hazard ratios of outcome in association with sex, age, and comorbidities in univariable and multivariable competing risk (death) model

	Acute pancreatitis		Cholangitis		Peptic ulcer bleeding		Cirrhosis	
Variable	Crude SHR^†^ (95% CI)	Adjusted SHR^‡^ (95% CI)	Crude SHR^†^ (95% CI)	Adjusted SHR^‡^ (95% CI)	Crude SHR^†^ (95% CI)	Adjusted SHR^‡^ (95% CI)	Crude SHR^†^ (95% CI)	Adjusted SHR^‡^ (95% CI)
Polycystic kidney disease	2.37 (1.97, 2.86)***	2.36 (1.95, 2.84)***	2.27 (1.82, 2.83)***	2.41 (1.93, 3.01)***	2.42 (2.18, 2.68)***	2.41 (2.17, 2.67)***	1.41 (1.18, 1.69)***	1.39 (1.16, 1.66)***
Gender (Women vs Men)	1.28 (1.07, 1.55)**	1.17 (0.97, 1.41)	1.29 (1.07, 1.55)**	1.17 (0.97, 1.41)	1.43 (1.29, 1.58)***	1.38 (1.25, 1.53)***	1.47 (1.25, 1.72)***	1.31 (1.12, 1.53)***
Age, per year	1.01 (1.01,1.01)***	1.01 (1.01, 1.01)***	1.01 (1.01, 1.02)***	1.01 (1.01, 1.01)***	1.04 (1.03, 1.04)***	1.03 (1.03,1.04)***	1.02 (1.02, 1.02)***	1.02 (1.02, 1.02)***
Baseline comorbidities (yes vs no)								
CKD	1.78 (1.49, 2.13)***	1.67 (1.28, 2.18)***	1.77 (1.48, 2.12)***	1.66 (1.27, 2.17)***	2.25 (2.05, 2.48)***	2.30 (2.01, 2.62)***	2.56 (2.20, 2.97)***	2.72 (2.20, 3.35)***
ESRD	1.53 (1.28, 1.83)***	1.08 (0.83, 1.41)	1.53 (1.28, 1.83)***	1.08 (0.83, 1.42)	1.73 (1.58, 1.91)***	0.92 (0.81, 1.05)	1.79 (1.55, 2.08)***	0.78 (0.64, 1.00)
GB stone disease	4.16 (3.37, 5.13)***	3.97 (3.21, 4.90)***	4.11 (3.33, 5.07)***	3.90 (3.16, 4.82)***	1.06 (0.90, 1.25)	-	1.15 (0.89, 1.50)	-
Hyperlipidemia	1.28 (0.98, 1.67)	-	1.28 (0.98, 1.67)	-	1.03 (0.88, 1.20)	-	0.80 (0.61, 1.04)	-
Obesity	-	-	-	-	1.80 (0.45, 7.20)	-	-	-
Diabetes	1.32 (1.06, 1.63)*	1.22 (0.98, 1.51)	1.32 (1.06, 1.63)*	1.22 (0.98, 1.51)	1.14 (1.02, 1.28)*	1.12 (1.00, 1.26)	0.91 (0.74, 1.10)	-
Alcoholism	5.66 (3.79, 8.44)***	5.62 (3.71, 8.50)***	5.39 (3.61, 8.05)***	5.38 (3.56, 8.14)***	1.87 (1.27, 2.76)**	1.84 (1.25, 2.72)***	4.96 (3.45, 7.15)***	5.10 (3.52, 7.39)***
Chronic HBV infection	1.37 (0.88, 2.14)	-	1.39 (0.89, 2.17)	-	1.07 (0.80, 1.42)	-	3.71 (2.89, 4.75)***	2.97 (2.31, 3.83)***
Chronic HCV infection	1.46 (0.94, 2.26)	-	1.47 (0.95, 2.27)	-	1.42 (1.11, 1.81)**	1.19 (0.93, 1.52)	4.48 (3.56, 5.64)***	3.46 (2.72, 4.39)***
NAFLD	1.60 (1.05, 2.43)*	1.18 (0.77, 1.81)	1.58 (1.04, 2.41)*	1.21 (0.79, 1.86)	1.03 (0.79, 1.34)	-	1.03 (0.67, 1.57)	-

† Crude SHR, relative subhazard ratio.

‡ aSHR: adjusted SHR. Covariables found significantly associated with outcome diseases in the univariable competing risk (death) model were further examined using the multivariable competing risk (death) model.

*p < 0.05, **p < 0.01, ***p < 0.001.

HBV: Hepatitis B virus; HCV: Hepatitis C virus; NAFLD: Nonalcoholic fatty liver disease.

Of 6031, 532 (8.8%) patients developed peptic ulcer bleeding with an incidence rate of 15.9 per 1000 person-years in the PKD cohort. The patients with PKD had a higher risk of peptic ulcer bleeding (aSHR 2.41 [95% CI, 2.17–2.67]) compared with the control cohort (Table 2). PKD, CKD, alcoholism, female sex, and increasing age were associated with an increased risk of peptic ulcer bleeding (Table 3).

The prevalence of chronic hepatitis and alcoholism was balanced over the study and control cohorts. A total of 154 (2.5%) patients developed cirrhosis in the PKD cohort. The patients with PKD had a 1.41-fold higher risk of cirrhosis (95% CI, 1.37–2.39) compared with the controls (Table 2). The presence of comorbidities such as alcoholism, chronic hepatitis B and C infection, CKD, PKD, female sex, and increasing age were associated with an increased risk of cirrhosis (Table 3).

### Secondary outcome: overall mortality

Kaplan–Meier analysis revealed an increased overall mortality in patients with PKD who developed acute pancreatitis and peptic ulcer bleeding compared with the controls (log-rank *p* < 0.05; Supplementary Figure 1).

## DISCUSSION

This investigation represents a national cohort study to evaluate major hepatointestinal complications other than common cystic manifestations in patients with PKD. When patients with PKD present with acute abdominal pain or febrile illness, clinicians often focus on excluding diagnoses that are more prevalent in PKD, such as cyst infection, cyst rupture or hemorrhage, and nephrolithiasis [7, 12]. However, patients still experience non-cystic intra-abdominal complications. Our findings corroborate the results from a UK epidemiologic study of 23,454 patients with PKD, which reported that patients with PKD exhibited higher rates of admission for biliary tract disease and serious liver complications [13]. Nevertheless, our results differed from theirs in two aspects. First, female predominance influenced the risk of both biliary disease and cirrhosis in our study, whereas in their study, a higher risk of biliary tract disease was observed in men than in women, and a higher risk of serious liver complications was observed in women than in men. Second, the presence of CKD, but not ESRD, reinforced hepatointestinal risks in our study. In brief, the exceeding risk of hepatointestinal manifestations was evident in our study, and two complications, acute pancreatitis and peptic ulcer bleeding, contributed to an increased overall mortality in patients with PKD.

### Acute pancreatitis and cholangitis in polycystic kidney disease

Acute, chronic, or recurrent pancreatitis and cholangitis occurred in patients with ADPKD [14–16]. Pancreatitis in patients with PKD is caused by the obstruction of the pancreatic duct by the pancreatic cystic compression, leading to duct distortions and exocrine dysfunction of the pancreas [17]. The frequency of pancreatic cysts ranged from 9%–36% in patients with PKD [18]. According to our review of the relevant literature, pancreatic cyst complications in PKD have not been determined. Although patients with pancreatic cysts are usually asymptomatic, they could present with pancreatitis or pancreatic malignancies [19, 20].

Common bile duct dilation is prevalent (40%) in patients with PKD [21]. Dranssart et al. observed intrahepatic bile duct abnormalities in 27.8% of the patients with PKD, which correlated with the results of hepatobiliary infection [22]. A case report described the presence of multifocal cystic dilatation of the intrahepatic bile ducts, such as Caroli's disease, in patients with PKD [23]. Hasegawa et al. reported a patient with diagnostic imaging findings indicating hepatobiliary abnormalities of Caroli's disease and cystic dilated biliary ducts with stones in endoscopic retrograde cholangiography, which led to recurrent cholangitis with hepatobiliary stones [24]. These patients may experience recurrent fever and septicemia. Appropriate diagnosis of cholangitis is necessary because antibiotics and biliary stone extraction are indicated whenever feasible.

Because recurrent infections and complications related to hepatobiliary system abnormalities can be associated with morbidity, we proposed that pancreatitis and cholangitis should be considered in the differential diagnosis of febrile or abdominal pain as well as cyst infection in patients with PKD.

### Peptic ulcer bleeding in polycystic kidney disease

Peptic ulcer bleeding is a medical condition that results in high morbidity. Risk factors for peptic ulcers include painkiller use, alcohol consumption, older age, and medical illnesses such as diabetes and ESRD [25–27]. Patients with PKD who presented with Budd–Chiari syndrome were prone to gastrointestinal bleeding from the varices or portal hypertensive gastropathy [28]. However, data on the risk of peptic ulcer bleeding in patients with PKD are lacking.

In our study cohort, 65.8% patients were older than 50 years, and patients with concomitant diabetes or ESRD were prevalent, which might be attributed to the association between PKD and peptic ulcer bleeding. Another possible explanation may be the use of analgesics, including acetaminophen and possibly intermittent short courses of NSAIDs or cyclooxygenase-2 inhibitors, in patients with chronic pain syndrome [29]. Additional potential confounders, such as *Helicobacter pylori* infection status, smoking, and painkiller or proton pump inhibitor consumption, should be considered in future research to elucidate the relationship between peptic ulcer bleeding and PKD. Nonetheless, our results represent the first quantification of this association and highlight the impact of peptic ulcer bleeding on overall mortality in patients with PKD.

### Liver cirrhosis in polycystic kidney disease

Until now, cirrhosis has been underrecognized in patients with PKD. Patients with PKD exhibit a relative loss of liver parenchyma in the late stages of the disease, with splenomegaly associated with liver cyst severity [30]. Some studies have reported patients with cirrhosis and have determined the mechanism of cirrhosis in patients PKD. Torres et al. reported patients with severe polycystic kidney or liver disease who developed Budd–Chiari syndrome characterized by ascites and hepatic venous outflow obstruction [28]. In patients with Budd–Chiari syndrome, the histological findings included centrilobular hepatocyte necrosis, obstructive portal venopathy, and cirrhosis [31, 32]. If left untreated, patients often die from intractable ascites, gastrointestinal bleeding, and liver failure. Ratcliffe et al. [33] reported an adult PKD patient with liver cysts who was diagnosed with portal hypertension complicated by bleeding esophageal varices. The authors proposed that hepatic dysfunction was caused by the replacement of hepatic parenchyma by cysts and compression of the portal vein by cysts contributing to portal hypertension.

Our result demonstrated an association between PKD and cirrhosis, and most patients with cirrhosis had concomitant chronic liver diseases. In addition, we demonstrated that alcoholism and concomitant chronic liver disease were independently associated with cirrhosis, which is consistent with other study results [34, 35]. Thus, we proposed that PKD patients with chronic liver disease or severe liver phenotype should be monitored for signs of liver decompensation. Early referral of these patients to hepatologists for evaluating the need for liver transplantation or combined liver or kidney transplantation is suggested [36].

### Study limitations

Our study is a retrospective registry-based study. Although the results are robust and the large sample size provides adequate statistical power for detecting the risk in the follow-up period, limitations should be addressed. First, patients with cirrhosis were identified using diagnostic codes recorded in the inpatient claims from the National Health Insurance Research Database (NHIRD) by clinicians based on imaging, the Child–Pugh score, and pathology. Detailed clinical information such as laboratory data, pathology staging, and radiographic findings was unavailable in this database. Nevertheless, catastrophic illness certification for cirrhosis was issued by hepatology specialists using the standard criteria after peer review.

Second, the severity of PLD in the PKD cohort was not demonstrated in our study because of database limitation. However, according to our review of the relevant literature, no study has established either the staging system for PLD severity or the association between PLD severity and clinical outcome in patients with PKD. Third, because early stage PKD is usually asymptomatic and could be underdiagnosed in the control cohort, outcome risk may have been overestimated in the control cohort. Fourth, this study could not provide CKD staging information because of database limitations. Alternatively, we categorized renal dysfunction into CKD and ESRD in our cohorts and observed different risk contribution to outcome diseases (Table 3).

## MATERIALS AND METHODS

### Data source

In this study, data were retrieved from the NHIRD, which is a nationwide electronic database containing the longitudinal medical records of beneficiaries enrolled in the National Health Insurance (NHI) program in Taiwan. The NHI program, launched on March 1, 1995, provides health care coverage to 99% of the population of Taiwan (approximately 23.75 million people). The NHIRD includes registries of enrolment files, diagnoses, procedures, drug prescriptions, and claims summaries of inpatients. Moreover, it includes the Registry of Catastrophic Illness Patients Database, which comprises 30 disease categories, including ESRD, cystic kidney disease, and cirrhosis. Beneficiaries of catastrophic illness certification are exempted from copayment for medical services. The International Classification of Diseases, Ninth Revision, Clinical Modification (ICD-9-CM) was used to define diseases. The diagnosis validity for diseases such as PKD, cirrhosis, acute pancreatitis, cholangitis, and peptic ulcer bleeding in medical publications from the NHIRD were credible [37–42]. Data validation demonstrated the high accuracy of the NHIRD in population-based studies [43, 44].

### Ethics statement

Patient records in the NHIRD are anonymized to protect patient privacy. This study was approved to fulfill the condition for exemption by the Institutional Review Board and the Ethics Committee of China Medical University (CMUH104-REC2-115-CR1). Informed patient consent was waived because the data used in this study were anonymized.

### Study design and patient selection

**Figure 1:**
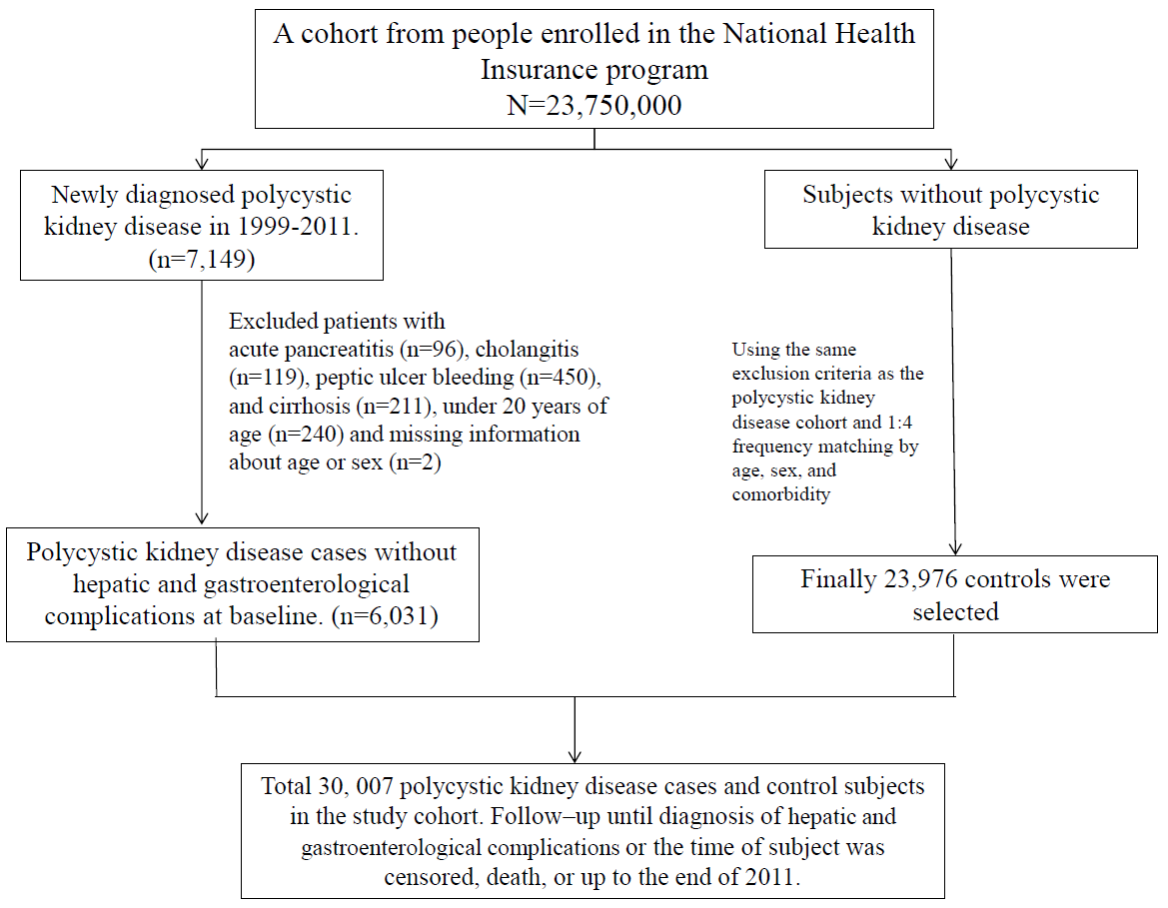
Flowchart of the cohort selection procedure

This was a retrospective cohort study; Figure 1 presents the study design. All inpatients with a primary diagnosis of PKD (ICD-9-CM codes 753.12 and 753.13) at admission between January 1, 2000, and December 31, 2010 were identified (*N* = 7149). Primary diagnosis of PKD included clinical symptoms, imaging studies, and family history. Among patients with PKD, 1118 were excluded on the basis of the following criteria: age less than 20 years; a history of acute pancreatitis (ICD-9-CM code 577.0), cholangitis (ICD-9-CM code 576.1), peptic ulcer bleeding (ICD-9-CM codes 531.0, 531.2, 531.4, 531.6, 532.0, 532.2, 532.4, 532.6, 533.0, 533.2, 533.4, and 533.6), and cirrhosis (ICD-9-CM codes 571.2, 571.5, and 571.6) prior to the index hospitalization; and missing information. A total of 6031 patients with PKD were enrolled as the study cohort. The control cohort comprised those who did not have a history of PKD, who were selected from the inpatient database by using the same exclusion criteria applied for the study cohort. The control cohort (*N* = 23,976) was PS-matched with the study cohort in a 1:4 ratio by sex, age, index date, and underlying comorbidities. The date of initial PKD diagnosis was defined as the index date.

**Figure 2:**
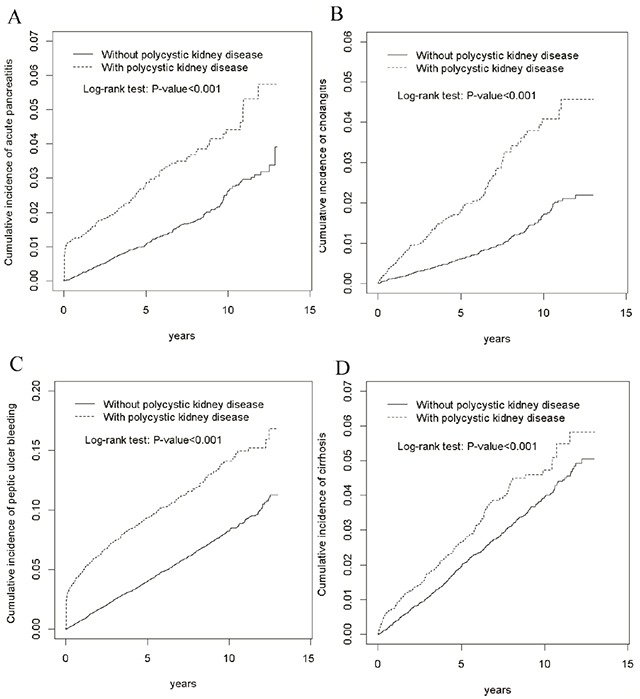
Cumulative incidence of acute pancreatitis **(A)**, cholangitis **(B)**, peptic ulcer bleeding **(C)**, and cirrhosis **(D)** in patients with and without polycystic kidney disease.

### Comorbidities

Demographic information of the study and control cohorts was obtained, including the underlying comorbid illnesses, which were diagnosed in previous inpatient claims or at least three successive outpatients claims before the index hospitalization date. We identified CKD (ICD-9-CM code 585) and ESRD (ICD-9-CM code 586) as indicators of PKD severity. Factors that could affect outcome, such as alcoholism (ICD-9-CM codes 291, 303, 305.00–305.3, 571.0, 571.1, and 571.3) and calculus of gallbladder (ICD-9 codes 574.0, 574.1, 574.2, 574.6, 574.7, 574.8, and 574.9), hyperlipidemia (ICD-9 codes 272.1–272.4, 272.8, and 272.9) for acute pancreatitis; and alcoholism, chronic hepatitis B virus infection (ICD-9 codes 070.22, 070.23, 070.32, 070.33, and V02.61), chronic hepatitis C virus infection (ICD-9 codes 070.41, 070.44, 070.51, 070.54, and V02.62), and nonalcoholic fatty liver disease (ICD-9 code 571.8) for cirrhosis were also evaluated.

### Outcomes

The primary outcomes were a diagnosis of acute pancreatitis, cholangitis, peptic ulcer bleeding, and cirrhosis during hospitalization in the follow-up period. Acute pancreatitis was mainly defined according to the Atlanta classification [45], with an inflammatory process of the pancreas with variable involvement of other local tissue and remote organ dysfunction. The classic triad of right upper quadrant pain, fever, and jaundice was used to define cholangitis. Primary diagnosis of peptic ulcer bleeding was made in patients who had undergone a panendoscopy (billing code: 47043B) during hospitalization. We excluded variceal hemorrhage (ICD-9 codes 456.0 and 456.20) related to cirrhosis. The follow-up period commenced from the index date until the occurrence of outcome, the date on which patients were censored because of withdrawal from the insurance program (e.g., death, immigration, and imprisonment), or end of the study period (Dec 31, 2011).

### Statistical analysis

Demographics and comorbidities were compared between the study and control cohorts by using a chi-square test for dichotomous variables and Student's *t* test for continuous variables. Multivariate analyses were used to model outcome diseases after adjustment for potential confounders. The cumulative incidence of the outcome was calculated using the Kaplan–Meier method, and Cox proportional hazard regression models were used to identify the risks of outcome. Because death was a potential confounding factor and could have biased the estimation of outcome risk, we analyzed a competing risk model to estimate the SHRs and 95% CIs of the outcome incidence in both cohorts by using the Fine and Gray method [46]. SAS (version 9.3; SAS Institute, Inc., Cary, NC, USA) was used for all statistical analyses. A two-sided *P* value of <.05 was considered statistically significant.

## CONCLUSION

Our study demonstrated that PKD is associated with major hepatointestinal complications. The development of acute pancreatitis and peptic ulcer bleeding had a negative impact on overall mortality in patients with PKD. Therefore, acute pancreatitis and cholangitis should be listed in the differential diagnosis of acute abdominal pain in patients with PKD. For anemia survey in patients with PKD, peptic ulcer bleeding should be considered.

## SUPPLEMENTARY MATERIALS FIGURES



## References

[1] Grantham JJ (2008). Clinical practice. Autosomal dominant polycystic kidney disease. N Engl J Med.

[2] Torres VE, Harris PC, Pirson Y (2007). Autosomal dominant polycystic kidney disease. Lancet.

[3] Schrier RW, McFann KK, Johnson AM (2003). Epidemiological study of kidney survival in autosomal dominant polycystic kidney disease. Kidney Int.

[4] Rahman E, Niaz FA, Al-Suwaida A, Nahrir S, Bashir M, Rahman H (2009). Analysis of causes of mortality in patients with autosomal dominant polycystic kidney disease: a single center study. Saudi J Kidney Dis Transpl.

[5] Perrone RD, Ruthazer R, Terrin NC (2001). Survival after end-stage renal disease in autosomal dominant polycystic kidney disease: contribution of extrarenal complications to mortality. Am J Kidney Dis.

[6] Bae KT, Zhu F, Chapman AB, Torres VE, Consortium for Radiologic Imaging Studies of Polycystic Kidney Disease (CRISP) (2006). Magnetic resonance imaging evaluation of hepatic cysts in early autosomal-dominant polycystic kidney disease: the Consortium for Radiologic Imaging Studies of Polycystic Kidney Disease cohort. Clin J Am Soc Nephrol.

[7] Grünfeld JP, Albouze G, Jungers P, Landais P, Dana A (1985). Liver changes and complications in adult polycystic kidney disease. Adv Nephrol Necker Hosp.

[8] Hogan MC, Norby SM (2010). Evaluation and management of pain in autosomal dominant polycystic kidney disease. Adv Chronic Kidney Dis.

[9] Gabow PA (1990). Autosomal dominant polycystic kidney disease--more than a renal disease. Am J Kidney Dis.

[10] Bajwa ZH, Gupta S, Warfield CA, Steinman TI (2001). Pain management in polycystic kidney disease. Kidney int.

[11] Suwabe T, Ubara Y, Mise K, Kawada M, Hamanoue S, Sumida K (2013). Quality of life of patients with ADPKD-Toranomon PKD QOL study: cross-sectional study. BMC Nephrol.

[12] Neuville M, Hustinx R, Jacques J, Krzesinski JM, Jouret F (2016). Diagnostic algorithm in the management of acute febrile abdomen in patients with autosomal dominant polycystic kidney disease. PLoS One.

[13] Judge PK, Harper CHS, Storey BC, Haynes R, Wilcock MJ (2017). Biliary tract and liver complications in polycystic kidney disease. J Am Soc Nephrol.

[14] Cano DA, Sekine S, Hebrok M (2006). Primary cilia deletion in pancreatic epithelial cells results in cyst formation and pancreatitis. Gastroenterology.

[15] Başar O, Ibiş M, Uçar E, Ertuğrul I, Yolcu OF (2006). Recurrent pancreatitis in a patient with autosomal-dominant polycystic kidney disease. Pancreatology.

[16] Yazdanpanah K, Manouchehri N, Hosseinzadeh E, Emami MH, Karami M (2013). Recurrent acute pancreatitis and cholangitis in a patient with autosomal dominant polycystic kidney disease. Int J Prev Med.

[17] Malka D, Hammel P, Vilgrain V, Fléjou JF, Belghiti J (1998). Chronic obstructive pancreatitis due to a pancreatic cyst in a patient with autosomal dominant polycystic kidney disease. Gut.

[18] Mikolajczyk AE, Te HS, Chapman AB (2017). Gastrointestinal manifestations of autosomal-dominant polycystic kidney disease. Clin Gastroenterol Hepatol.

[19] Sato Y, Mukai M, Sasaki M, Kitao A, Yoneda N, Kobayashi D, Imamura Y, Nakanuma Y (2009). Intraductal papillary–mucinous neoplasm of the pancreas associated with polycystic liver and kidney disease. Pathol Int.

[20] Niv Y, Turani C, Kahan E, Fraser GM (1997). Association between pancreatic cystadenocarcinoma, malignant liver cysts, and polycystic disease of the kidney. Gastroenterology.

[21] Ishikawa I, Chikamoto E, Nakamura M, Asaka M, Tomosugi N (1996). High incidence of common bile duct dilatation in autosomal dominant polycystic kidney disease patients. Am J Kidney Dis.

[22] Dranssart M, Cognet F, Mousson C, Cercueil JP, Rifle G, Krause D (2002). MR cholangiography in the evaluation of hepatic and biliary abnormalities in autosomal dominant polycystic kidney disease: study of 93 patients. J Comput Assist Tomogr.

[23] Mousson C, Rabec M, Cercueil JP, Virot JS, Hillon P, Rifle G (1997). Carolit disease and autosomal dominant polycystic kidney disease: a rare association?. Nephrol Dial Transplant.

[24] Hasegawa E, Sawa N, Hoshino J, Suwabe T, Hayami N, Yamanouchi M (2016). Recurrent cholangitis in a patient with autosomal dominant polycystic kidney disease (ADPKD) and caroli's disease. Intern Med.

[25] Wang YR, Richter JE, Dempsey DT (2010). Trends and outcomes of hospitalizations for peptic ulcer disease in the United States, 1993 to 2006. Ann Surg.

[26] Schimke K, Chubb SA, Davis WA, Phillips P, Davis TM (2009). Antiplatelet therapy, Helicobacter pylori infection and complicated peptic ulcer disease in diabetes: the Fremantle Diabetes Study. Diabet Med.

[27] Wu CY, Wu MS, Kuo KN, Wang CB, Chen YJ, Lin JT (2011). Long-term peptic ulcer rebleeding risk estimation in patients undergoing haemodialysis: a 10-year nationwide cohort study. Gut.

[28] Torres VE, Rastogi S, King BF, Stanson AW, Gross JB (1994). Hepatic venous outflow obstruction in autosomal dominant polycystic kidney disease. J Am Soc Nephrol.

[29] Henrich WL, Agodoa LE, Barrett B, Bennett WM, Blantz RC (1996). Analgesics and the kidney: summary and recommendations to the Scientific Advisory Board of the National Kidney Foundation from an Ad Hoc Committee of the National Kidney Foundation. Am J Kidney Dis.

[30] Hogan MC, Abebe K, Torres VE, Chapman AB, Bae KT (2015). Liver involvement in early autosomal-dominant polycystic kidney disease. Clin Gastroenterol Hepatol.

[31] Gonzalez RS, Gilger MA, Huh WJ, Washington K (2017). The spectrum of histologic findings in hepatic outflow obstruction. Arch Pathol Lab Med.

[32] Cazals-Hatem D, Vilgrain V, Genin P, Denninger MH, Durand F, Belghiti J (2003). Arterial and portal circulation and parenchymal changes in Budd-Chiari syndrome: a study in 17 explanted livers. Hepatology.

[33] Ratcliffe PJ, Reeders S, Theaker JM (1984). Bleeding oesophageal varices and hepatic dysfunction in adult polycystic kidney disease. Br Med J (Clin Res Ed).

[34] Yang JD, Mohamed HA, Cvinar JL, Gores GJ, Roberts LR, Kim WR (2016). Diabetes mellitus heightens the risk of hepatocellular carcinoma except in patients with hepatitis C cirrhosis. Am J Gastroenterol.

[35] Goh GB, Pan A, Chow WC, Yuan JM, Koh WP (2017). Association between diabetes mellitus and cirrhosis mortality: the singapore chinese health study. Liver Int.

[36] Mehrabi A, Fonouni H, Ayoub E, Rahbari NN, Müller SA, Morath Ch, Seckinger J, Sadeghi M, Golriz M, Esmaeilzadeh M, Hillebrand N, Weitz J, Zeier M (2009). A single center experience of combined liver kidney transplantation. Clin Transplant.

[37] Yu TM, Chuang YW, Yu MC, Chen CH, Yang CK, Huang ST (2016). Risk of cancer in patients with polycystic kidney disease: a propensity-score matched analysis of a nationwide, population-based cohort study. Lancet Oncol.

[38] Hung TH, Tsai CC, Hsieh YH, Tsai CC, Tseng CW (2016). The effect of the first spontaneous bacterial peritonitis event on the mortality of cirrhotic patients with ascites: a nationwide population-based study in taiwan. Gut Liver.

[39] Su TH, Hu TH, Chen CY, Huang YH, Chuang WL, Lin CC (2016). Four-year entecavir therapy reduces hepatocellular carcinoma, cirrhotic events and mortality in chronic hepatitis B patients. Liver Int.

[40] Chen HJ, Wang JJ, Tsay WI, Her SH, Lin CH, Chien CC (2017). Epidemiology and outcome of acute pancreatitis in end-stage renal disease dialysis patients: a 10-year national cohort study. Nephrol Dial Transplant.

[41] Liang CM, Hsu CN, Tai WC, Yang SC, Wu CK, Shih CW, Taiwan Acid-Related Disease (TARD) Study Group (2016). Risk factors influencing the outcome of peptic ulcer bleeding in chronic kidney disease after initial endoscopic hemostasis: A nationwide cohort study. Medicine (Baltimore).

[42] Wei CY, Chung TC, Chen CH, Lin CC, Sung FC, Chung WT (2014). Gallstone disease and the risk of stroke: a nationwide population-based study. J Stroke Cerebrovasc Dis.

[43] Cheng CL, Chien HC, Lee CH, Lin SJ, Yang YH (2015). Validity of in-hospital mortality data among patients with acute myocardial infarction or stroke in National Health Insurance Research Database in Taiwan. Int J Cardiol.

[44] Chen HL, Hsiao FY (2014). Risk of hospitalization and healthcare cost associated with Diabetes Complication Severity Index in Taiwan's National Health Insurance Research Database. J Diabetes Complications.

[45] Bradley EL A clinically based classification system for acute pancreatitis. Arch Surg 1993.

[46] Fine JP, Gray RJ (1999). A proportional hazards model for the subdistribution of a competing risk. J Am Stat Assoc.

