# Supplementary material for: Hepatointestinal complications in polycystic kidney disease
Source: Oncotarget. 2017 Sep 15;8(46):80971–80. doi: 10.18632/oncotarget.20901 (PMC5655254; doi:10.18632/oncotarget.20901)
Supplement: Supplementary file 1 [file oncotarget-08-80971-s001.pdf]

Hepatointestinal complications in polycystic kidney disease

SUPPLEMENTARY MATERIALS

A

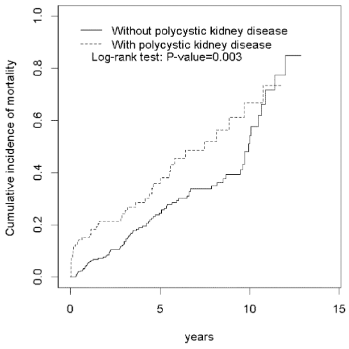

With polycystic kidney disease, No. At risk	160	29	5
Acute pancreatitis events	0	33	9
Without polycystic kidney disease, No. At risk	302	116	15
Acute pancreatitis events	0	53	21

C

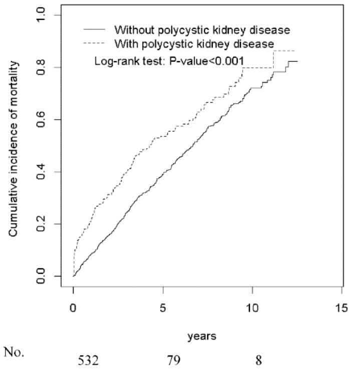

With polycystic kidney disease, No. At risk	532	79	8
Peptic ulcer bleeding events	0	166	26
Without polycystic kidney disease, No. At risk	1068	361	50
Peptic ulcer bleeding events	0	323	124

B

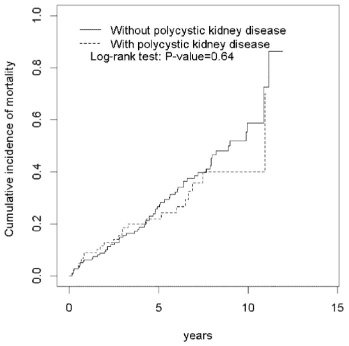

With polycystic kidney disease, No. At risk	113	36	2
Cholangitis events	0	18	6
Without polycystic kidney disease, No. At risk	188	82	11
Cholangitis events	0	38	23

D

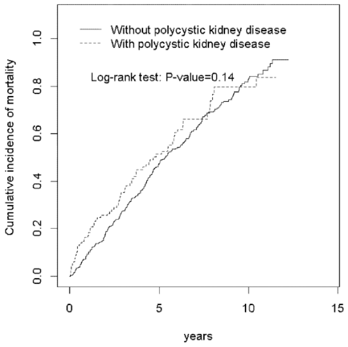

With polycystic kidney disease, No. At risk	154	41	5
Cirrhosis events	0	62	16
Without polycystic kidney disease, No. At risk	501	167	22
Cirrhosis events	0	194	77

Supplementary Figure 1: Cumulative incidence of mortality after developing acute pancreatitis **(A)**, cholangitis **(B)**, peptic ulcer bleeding **(C)**, and cirrhosis **(D)** in patients with and without polycystic kidney disease.
